# Efficacy and Safety of Direct Hemoperfusion Using Polymyxin B-Immobilized Polystyrene Column for Patients With COVID-19: Protocol for an Exploratory Study

**DOI:** 10.2196/37426

**Published:** 2022-11-16

**Authors:** Junko Terada-Hirashima, Shinyu Izumi, Daisuke Katagiri, Yukari Uemura, Ayako Mikami, Wataru Sugiura, Shinji Abe, Arata Azuma, Haruhito Sugiyama

**Affiliations:** 1 Center for Clinical Sciences National Center for Global Health and Medicine Tokyo Japan; 2 Department of Respiratory Medicine Center Hospital of the National Center for Global Health and Medicine Tokyo Japan; 3 Department of Nephrology Center Hospital of the National Center for Global Health and Medicine Tokyo Japan; 4 Department of Respiratory Medicine Tokyo Medical University Hospital Tokyo Japan; 5 Department of Pulmonary Medicine and Oncology Graduate School of Medicine Nippon Medical School Tokyo Japan

**Keywords:** polymyxin B-immobilized fiber column, PMX, diffuse alveolar damage, DAD, COVID-19, pneumonia, fibrinogenolysis, systemic inflammatory response syndrome, lung disease, lung damage, pulmonary, treatment, prospective intervention, health information, treatment information, therapy, COVID-19 therapy

## Abstract

**Background:**

Polymyxin B-immobilized fiber column (PMX; Toraymyxin column) was approved for the relief of systemic inflammatory response syndrome caused by bacterial infection or endotoxemia. PMX reduces lung damage by removing leukocytes and cytokines in addition to endotoxin removal in the setting of idiopathic pulmonary fibrosis. Acute exacerbation of interstitial pneumonia pathologically presents with diffuse alveolar damage (DAD). PMX direct hemoperfusion (PMX-DHP) demonstrated efficacy, improving oxygenation. The SARS-CoV-2 virus causes COVID-19, which emerged in December 2019. The condition may become severe about 1 week after onset, and respiratory failure rapidly develops, requiring intensive care management. A characteristic of COVID-19–related severe pneumonia is ground-glass opacities rapidly progressing in both lungs, which subsequently turn into infiltrative shadows. This condition could be classified as DAD. As for the congealing fibrinogenolysis system, D-dimer, fibrin/fibrinogen degradation product quantity, and prolonged prothrombin time were significant factors in nonsurviving COVID-19 cases, associated with aggravated pneumonia. Clinical trials are being conducted, but except for remdesivir and dexamethasone, no treatments have yet been approved. COVID-19 aggravates with the deterioration of oxygen saturation, decrease in lymphocytes, and the occurrence of an abnormal congealing fibrinogenolysis system, leading to diffuse lung damage. Once the condition transitions from moderate to severe, it is necessary to prevent further exacerbation by providing treatment that will suppress the aforementioned symptoms as soon as possible.

**Objective:**

This study aims to access treatment options to prevent the transition from acute exacerbation of interstitial pneumonia to DAD. The mechanism of action envisioned for PMX-DHP is to reduce congealing fibrinogenolysis system abnormalities and increase oxygenation by removing activated leukocytes and cytokines, which are risk factors for the aggravation of COVID-19–related pneumonia.

**Methods:**

We will conduct a multicenter, prospective, intervention, single-group study to evaluate the efficacy and safety of direct hemoperfusion using PMX-DHP for patients with COVID-19. Efficacy will be evaluated by the primary end point, which is the rate of Ordinal Scale for Clinical Improvement after PMX-DHP of at least 1 point from a status of 4, 5, or 6 on day 15. The effect of PMX-DHP will be estimated by setting a control group with background factors from non–PMX-DHP patients enrolled in the COVID-19 registry. This study will be carried out as a single-group open-label study and will be compared with a historical control. The historical control will be selected from the COVID-19 registry according to age, gender, and severity of pneumonia.

**Results:**

The study period is scheduled from September 28, 2020, through April 30, 2023. Patient enrollment was scheduled from the Japan Registry of Clinical Trials publication for March 31, 2022. Data fixation is scheduled for October 2022, with the publication of the results by March 2023.

**Conclusions:**

From a clinical perspective, PMX-DHP is expected to become an adjunctive therapy to address unmet medical needs and prevent the exacerbation from moderate to severe acute respiratory distress syndrome in COVID-19 cases.

**International Registered Report Identifier (IRRID):**

DERR1-10.2196/37426

## Introduction

Regarding polymyxin B-immobilized column (PMX), Toraymyxin was approved in October 1993 for the relief of systemic inflammatory response syndrome caused by gram-negative bacterial infection or endotoxemia by “selectively adsorbing and removing endotoxin in the blood by whole blood hemoperfusion.” It has been reported to be effective against acute lung injury/acute respiratory distress syndrome caused by sepsis, which is an indicator [1-4]. This is thought to be because PMX reduces lung damage by removing leukocytes and cytokines in addition to endotoxin removal. In terms of treatment results, idiopathic pulmonary fibrosis (IPF), which has been the subject of previous clinical studies, is the most common idiopathic interstitial pneumonia (accounts for about 50% of idiopathic interstitial pneumonia worldwide) and has a chronic and progressive course. Once advanced fibrosis progresses, irreversible honeycombing occurs, leading to an extremely poor prognosis [5,6]. The total number of Japanese patients with IPF is estimated to be at least around 10,000 [7]. Acute exacerbation of interstitial pneumonia pathologically presents with diffuse alveolar damage (DAD). In recent years, reports on PMX (Toraymyxin by Toray Industries Inc) direct hemoperfusion (PMX-DHP) for this acute exacerbation have mentioned its efficacy, which includes the improvement of oxygenation [8-15]. A study of 73 IPF cases with acute exacerbation has noted the improvement of oxygenation upon carrying out PMX-DHP [14]. Further, an exploratory study on the efficacy and safety of direct hemoperfusions using PMX-DHP for patients with IPF with acute exacerbation was conducted from 2014 to 2019 for the acute exacerbation of IPF. In this study, the survival rate of 20 enrolled cases 4 weeks after PMX-DHP was 65%, which far exceeded the 10% to 40% threshold survival rate in previous reports [15,16] and exceeded the expected upper limit of the survival rate of 60%. Moreover, the result was well above the expected lower limit of the 95% CI, which was 39%.

SARS-CoV-2 was identified as the causative virus of an unknown pneumonia (COVID-19) that emerged in Wuhan City, Hubei Province, China in December 2019. Depending on the case, the condition become severe about 1 week after onset, and respiratory failure rapidly develops, requiring intensive care management [17]. Patients may have markedly impaired oxygenation early in the course of the disease, which is said to be due to increased activity of the coagulation system; plasminogen activator inhibitor-1 expression is increased in older adults and in people with hypertension, obesity, and diabetes, which are risk factors for COVID-19; and in severe cases of COVID-19, STAT3 and plasminogen activator inhibitor-1 activation is escalated, leading to catastrophic consequences. A characteristic of COVID-19–related severe pneumonia cases is ground-glass opacities rapidly progressing in both lungs visible in chest computed tomography findings [18]. With subsequent progression toward severity, these opacities turn into infiltrative shadows [19]. This condition could be classified as DAD [20]. According to the analysis results of 191 COVID-19 cases in Wuhan, 100% of nonsurviving cases have sepsis while 93% have acute respiratory distress syndrome. The three most relevant risk factors in the early stage were age, Sequential Organ Failure Assessment score, and 1 μg/mL or higher D-dimer [21]. As for the congealing fibrinogenolysis system, according to a different report, D-dimer, fibrin and fibrinogen degradation product, and prolonged prothrombin time were significant factors in nonsurviving COVID-19 cases, suggesting that the effects of the congealing fibrinogenolysis system are associated with the aggravation of pneumonia [22]. As of February 2021, clinical trials and clinical studies are being conducted with several existing treatments, but except for remdesivir and dexamethasone, no drugs or treatments have yet been approved by the Japanese regulatory authorities.

As previously mentioned regarding the clinical significance of PMX-DHP, we believe that COVID-19 aggravates with the rapid deterioration of oxygen saturation, decrease in lymphocytes, and the occurrence of an abnormal congealing fibrinogenolysis system, leading to complex diffuse lung damage. Virus growth suppression caused by antiviral action can be expected if the initial growth rate is suppressed; however, once the condition transitions from moderate to severe, it is most necessary to address unmet medical needs to prevent further exacerbation by providing treatment that will suppress the aforementioned symptoms as soon as possible and prevent the transition to DAD. Simply put, we believe that, in addition to antiviral drugs, it is most necessary to acquire treatment options to prevent progression from rapidly worsening interstitial pneumonia to DAD. The complex mechanism of action envisioned for PMX-DHP is to reduce congealing fibrinogenolysis system abnormalities and increase oxygenation by removing activated leukocytes and cytokines, which are risk factors for the aggravation of COVID-19–related pneumonia. Therefore, from a clinical perspective, PMX-DHP is expected to become an adjunctive therapy to address unmet medical needs and prevent the progression from moderate to severe condition. The scientific rationale of this study is to carry out PMX-DHP, in addition to regular medical care, for patients with COVID-19 and to aggregate and analyze the acquired information. This study will help determine PMX-DHP treatment options in the medical setting by quickly collecting and publishing information on patient background and on the efficacy and safety of treatment by PMX-DHP.

## Methods

### Study Design

We will conduct a multicenter, prospective, intervention, single-group study to evaluate the efficacy and safety of direct hemoperfusion using PMX-DHP for patients with COVID-19. Efficacy will be evaluated by the primary end point, which is the rate of Ordinal Scale for Clinical Improvement after PMX-DHP of at least 1 point from a status of 4, 5, or 6 on day 15, based on previous studies for COVID-19 treatment (Table 1) [23]. Further, safety will be confirmed by the incidence of serious adverse events and problems. The effect of PMX-DHP will be estimated by setting a control group with background factors from non–PMX-DHP patients enrolled in the COVID-19 registry at participating facilities. Since this study will carry out PMX-DHP, setting up a sham group is difficult in terms of ethics because the therapy is relatively invasive. As such, this study will be carried out as a single-group open-label study and will be compared with a historical control. The historical control will be selected from the COVID-19 registry according to age, gender, and severity of pneumonia.

**Table 1:** Purpose and end points.

Purpose			End points		Validity and reason for selection of end points
**Primary**					
	To evaluate the efficacy of PMX-DHP^a^	Improvement rate (decrease) of 1 point or more from status 4, 5, or 6 on day 15 based on the eight-category evaluation		Because it was the primary end point in previous studies for COVID-19 treatment	
**Secondary**					
	To evaluate the efficacy and safety of PMX-DHP	Pathological improvement rate (status 1, 2, and 3 in the aforementioned eight categories) from the start of PMX^b^. PaO2/FiO2 improvement on day 4 and day 8 Changes in cytokines, coagulation markers, and urinary biomarkers Occurrence of serious adverse events and problems Invasive mechanical ventilation avoidance rate and duration of use ECMO^c^ avoidance rate and duration of use Mortality rate		Evaluation of respiratory function, safety, and efficacy of PMX-DHP	

^a^PMX-DHP: polymyxin B-immobilized fiber column direct hemoperfusion.

^b^PMX: polymyxin B-immobilized fiber column.

^c^ECMO: extracorporeal membrane oxygenation.

### Eligibility Criteria and Recruitment

All of the following inclusion criteria must be met to participate in this study: dyspnea unexplainable by other diseases (heart failure, renal failure, etc), diagnosis with SARS-CoV-2 infection through polymerase chain reaction or loop-mediated isothermal amplification within 1 week, at least one lung opacity on imaging suggestive of consolidation, P/F ratio of 300 or below or an SpO2 of 93% or below (indoor air), being hospitalized, requiring supplemental oxygen, requiring nasal high-flow oxygen therapy or noninvasive mechanical ventilation or requiring invasive mechanical ventilation, 16 years or older at the time of consent, and written consent. Consent is obtained from patients with pneumonia who are hospitalized for COVID-19 and who may meet the eligibility criteria.

Consent is obtained in writing from the individual. Informed consent will be obtained from a representative of the patient if the patient is a minor or objectively judged to have insufficient understanding of the subject due to a serious illness. The representative will be selected from the patient’s spouse, parents, siblings, children/grandchildren, grandparents, relatives living in the same household, or close relatives of the patient.

The following tests, observations, and evaluation will be performed as part of the screening test: vital information (date of birth, gender, height, weight, presence of oxygen therapy); clinical history (onset: date of onset, symptoms), treatment history (type, treatment period of therapeutic drugs/therapy), medical history, complications, allergies, date of admission, date of polymerase chain reaction positive result, concomitant drug confirmation (medication currently being taken by the patient shall be confirmed at the time of the screening), vital signs, blood test (hematological test, blood biochemical test), urinalysis, and imaging test (presence of pneumonia complications).

As a result of the screening, if the patient meets the enrollment criteria stated in the eligibility criteria, they will be enrolled to the study, and PMX-DHP will commence. Otherwise, the patient fails the screening and will no longer participate in the study.

The following exclusion criteria will be used: severe progression of multiple organ failure; P/F ratio of 100 or below; extracorporeal membrane oxygenation (ECMO); hospitalization for more than 15 days; platelet count of 20,000/μL or less; cytotoxic or biological treatments (anti-interleukin [IL]–1, anti-IL-6 [tocilizumab or sarilumab], T cell or B cell targeted treatment [rituximab, etc], tyrosine kinase inhibitor or interferon) within 4 weeks prior to consent; treatment with tumor necrosis factor inhibitor within 2 weeks prior to consent; treatment with convalescent plasma or intravenous immunoglobulin for COVID-19; and consideration by the principal investigator or subinvestigator to be unfit to participate in this study.

In case of nonparticipation in the study, administration of remdesivir, dexamethasone, and tocilizumab will be considered if indicated and not already administered. Furthermore, systemic management including oxygen therapy will be performed for deterioration of respiratory function.

A schematic illustration of the study design is presented in Figure S1 in Multimedia Appendix 1.

### Sample Size Estimation

In this study, the efficacy of PMX-DHP will be evaluated by comparing the improvement rate (decrease) of 1 point or more from status 4, 5, or 6 on day 15 based on the eight-category evaluation with the COVID-19 registry. In a paper that reported on the compassionate use of remdisivir for COVID-19 cases [23], of 51 cases who were under invasive ventilation, noninvasive oxygen support, or low flow oxygen (corresponds to the status 4, 5, and 6 in this study), improvement by at least 1 stage was noted in 34 cases (approximately 57%; median follow-up period: 18 days). Further, in a randomized clinical trial investigating the effect of lopinavir-ritonivar on COVID-19 about 38% (75/199) of cases recovered (improved by 2 points or more on a 6-point scale) 14 days after randomization [24]. Based on the aforementioned, we assume that approximately 50% to 60% of non–PMX-DHP COVID-19 cases will improve by 1 point. On the other hand, although there is insufficient data on the expected effects of PMX-DHP, we can consider PMX-DHP effective if the improvement rate of cases in this study is 1.5 times that of the non–PMX-DHP cases.

We set a 2-sided type I error at 10%. When the improvement rate of non–PMX-DHP cases is approximately 55%; if there are 30 cases in this study; and the control group is set at 30, 60, and 90 cases, the detection power would be approximately 65%, 78%, and 83%, respectively. Similarly, if the improvement rate of non–PMX-DHP cases is approximately 50%, the detection power would be 54%, 67%, and 72%, respectively. If the improvement rate is approximately 60%, the detection power would be 78%, 90%, and 94%, respectively. However, when compared with control cases, the detection power fluctuates due to the adjustment in the confounding factor.

### Intervention: Medical Device, Protocol, and Combination Therapy

The medical device used is an adsorptive blood-purifying device (product name Toraymyxin, model PHX-20R, approval 20500BZZ00926000). This blood-purifying device selectively adsorbs and removes blood endotoxin by whole blood hemoperfusion. This product is intended to improve the pathological condition by treating patients with severe pathological conditions associated with endotoxemia or thought to be due to gram-negative bacterial infections.

The appearance of this product is shown in Table 2 and Figure S2 in Multimedia Appendix 2.

**Table 2:** Structure.

	Toraymyxin (model PHX-20R)
Length (mm)	225
Maximum diameter (mm)	63
Body diameter (mm)	49
Blood volume (mL)	135 (±5)

This product is sealed one by one in a sterilized bag and packed in a box. Information about storage, delivery, disposal, potential serious side effects, and caution for concomitant treatment can be obtained at the manufacturer’s website [25]. In general, an adverse event is an undesirable symptom, sign, disease, or abnormal laboratory test value that occurs in a patient regardless of their causal relationship with the study or the pharmaceuticals used in this study. An adverse event occurs after the start of treatment. An adverse event will be considered a serious adverse event if the principal investigator or subinvestigator rules that the following criteria have been met: death or risk of leading to death, events that require hospitalization at a medical institution for treatment or require the extension of hospitalization, impairment or risk of leading to impairment, or congenital diseases or abnormalities that could be inherited by later generations.

### Dosage

While administering an anticoagulant drug (nafamostat mesilate: approximately 30-40 mg/hr or heparin approximately 40-60 U/kg one shot + approximately 40-60 U/kg/h), 1 Toraymyxin at a flow rate of 60-120 mL per minute should be administered for 3-6 hours (maximum of 24 hours), and at least 2 doses (maximum 3) should be used. The follow-up will be carried out up to 4 weeks after the end of PMX-DHP. PMX-DHP does not have to be conducted in consecutive days, but the interval between each session must be as short as possible.

The equipment operation for direct hemoperfusion will go as follows (Multimedia Appendix 3):

1. Prepare the extracorporeal circulation device (blood pump, anticoagulant infusion pump, monitoring of arterial and venous pressure of the inlet and outlet pressure of this product)
2. Precautions before use (inspection of exterior, confirmation of sterilization)
3. Cleaning and priming
4. Circulation:
Insert a double lumen catheter into the patient’s femoral vein or internal jugular vein to serve as the site for blood access
Using a blood pump, perform whole blood hemoperfusion at a flow rate of 60-120 mL per minute, which will be determined according to the patient’s condition
When using this product, anticoagulants (nafamostat mesylate, heparin) are administered by continuous infusion from the blood circuit on the blood removal side.
Duration of extracorporeal circulation is 3 to 24 hours per piece
The maximum working pressure of this product is 66 kPa (500 mHg). Note that the pressure difference between the inlet and outlet of this product may increase due to internal clogging caused by the formation of blood clots.

Heparin or nafamostat will be used in the anticoagulant therapy to be performed for direct hemoperfusion associated with PMX-DHP. Moreover, since the target patients of this treatment are within the scope of heparin and nafamostat, the anticoagulation treatment is not considered as the target treatment of this study.

There are no restrictions on the antiviral treatment for COVID-19; however, in principle, the antiviral treatment will not be changed during the study period.

Concomitant use with cytotoxic or biological treatments (anti-IL-1, anti-IL-6 [tocilizumab or sarilumab], T cell or B cell targeted treatment [rituximab, etc], tyrosine kinase inhibitor or interferon), tumor necrosis factor inhibitor, convalescent plasma, and intravenous immunoglobulin administration for COVID-19 are prohibited. Concomitant use of steroids, including dexamethasone and tocilizumab, is acceptable.

### Statistical Analysis

The main purpose of this study is to verify the efficacy in PMX-DHP cases compared to the control in the COVID-19 registry for the primary end point. Particularly, the efficacy of PMX-DHP will be evaluated by comparing the improvement rate (decrease) of 1 point or more from status 4, 5, or 6 on day 15 based on the eight-category evaluation with the COVID-19 registry.

1. Not hospitalized with resumption of normal activities
2. Not hospitalized but unable to resume normal activities
3. Hospitalized not requiring supplemental oxygen
4. Hospitalized requiring supplemental oxygen
5. Hospitalized requiring nasal high-flow oxygen therapy, noninvasive mechanical ventilation, or both
6. Requiring invasive mechanical ventilation
7. Requiring ECMO
8. Death

The status and the percentage of PMX-DHP cases at each evaluation time will be calculated, and the number and percentage of people whose day 15 status improved by 1 or more points from day 1 will be calculated. Furthermore, the following analysis will be planned for comparison with control cases.

Bias is included when comparing the rate of improvement of control cases for 15 days from the time of hospitalization against cases that underwent PMX-DHP several days after hospitalization. Therefore, the number of days from hospitalization to the date of PMX-DHP administration will be calculated for each case.

The effect of PMX-DHP will be estimated using the propensity score based on the data, which integrates this study and the registry. The propensity score will be created based on age, gender, body temperature, etc, and analysis methods such as matching will be used. A significance level on both sides of 5% will be used.

The following results will be calculated for cases enrolled in this study:

- Pathological improvement rate on day 15 (status 1, 2, and 3 in the eight categories) from the start of PMX. The number and percentage of people who reached status 1, 2, and 3 on day 15 at a 95% CI will be calculated. Furthermore, the same items will be calculated on day 29.
- P/F improvement on day 4 and 8. The descriptive statistics of P/F on day 1, day 4, and day 8 will be calculated and presented on a transition chart.
- Changes in cytokines, coagulation markers, and urinary biomarkers. The descriptive statistics of clinical laboratory test values that take continuous values will be calculated. For laboratory findings, which are classification variables, the number and proportions at each level will be calculated at each point in time.
- Invasive mechanical ventilation avoidance rate and duration of use. The number and proportion of people who underwent invasive mechanical ventilation between day 15 and 29 will be calculated.
- ECMO avoidance rate and duration of use. The number and proportion of people who underwent ECMO between day 15 and 29 will be calculated.

Similar to the primary end point, the efficacy of PMX will be evaluated for the aforementioned secondary end points using propensity scores based on data integrated with the enrolled cases in the registry.

The safety end point is the incidence and rate of adverse events. Multiplicity will not be adjusted in the analysis of safety end points. In the estimation of the ratio to the number of cases and presence or absence of occurrence, an exact 95% CI for the binomial distribution will be calculated for each group.

In addition to clinical laboratory items, continuous quantity data will be tabulated by group through descriptive statistics, while classification variables will be tabulated through the number of people or rate, among other appropriate methods.

Furthermore, PMX malfunctions will also be tabulated. Subgroup analysis will be performed by status and age at the time of enrollment.

### Ethics Approval

This study will be reviewed by the Institutional Review Board for Clinical Research of National Center for Global Health and Medicine (certification CRB3200011).

## Results

The study period is scheduled from September 28, 2020, the date of publication in the Japan Registry of Clinical Trials (jRCT), through April 30, 2023. Patient enrollment is scheduled from the jRCT publication to March 31, 2022. Data fixation is scheduled for October 2022, with publication of results by March 2023.

## Discussion

We are conducting an interventional trial to evaluate the efficacy of PMX in moderately to severely ill patients with COVID-19 requiring oxygenation, believing that PMX may be useful in the treatment of COVID-19 by removing inflammatory cytokines and improving the coagulation system.

### Anticipated Findings

Participation in this study may improve respiratory function and homeostasis of the congealing fibrinogenolysis system by receiving PMX-DHP. PMX-DHP can be performed in parallel with treatment using antiviral drugs, etc, that are expected to have a therapeutic effect on COVID-19, which may be beneficial to the patients. In addition to the direct benefits to the patients, if PMX-DHP is recognized as a standard treatment through this study, it may lead to the reduction of burden on patients with COVID-19 and reduction of medical expenses for society. Furthermore, patients may indirectly benefit once the research results concerning COVID-19 treatment help rehabilitate society. In this study, all eligible patients receive PMX-DHP but with the risk of pain, bleeding, and infection associated with catheter insertion.

Furthermore, the same dangers and discomfort similar to regular medical treatment may occur as a result of intravenous blood sampling and radiological imaging, for example, pain and discomfort associated with blood sampling and exposure to radiation imaging. The blood to be collected per sampling in this study will be 30 mL, which is medically acceptable, but since the frequency of blood collection is higher than in normal medical care, the burden on the patient may increase. Since blood will be collected for a total of 5 times, 150 mL of blood or more will be collected throughout the entire period of the study compared to regular medical care.

### Limitations

The major limitations of this study are that it is an open-label trial, where the information is not withheld from trial participants (both the researchers and participants know that PMX therapy is being administered), and it is not a randomized parallel study, so the patients will be allocated to different groups in a nonrandom way. We plan to use the COVID-19 registry as a control group, but selection bias should be interpreted with caution [26]. Finally, the severity of COVID-19 depends on the mutant strain. Depending on the future epidemic, fewer cases may be severe, making it more difficult to incorporate.

### Dissemination Plan

The results will be presented at national and international academic meetings, and submitted to peer-reviewed journals for publications.

## Appendix

Multimedia Appendix 1 Schematic overview of the study design. PMX-DHP: Polymyxin B-immobilized fiber column direct hemoperfusion.

Multimedia Appendix 2 External view of Toraymyxin.

Multimedia Appendix 3 Clinical example of Toraymyxin. PMX: polymyxin B-immobilized fiber column.

## Abbreviations

DAD: diffuse alveolar damage
ECMO: extracorporeal membrane oxygenation
IL: interleukin
IPF: idiopathic pulmonary fibrosis
jRCT: Japan Registry of Clinical Trials
PMX: polymyxin B-immobilized fiber column
PMX-DHP: polymyxin B-immobilized fiber column direct hemoperfusion
