# Supplementary figures and images for: Efficacy and Safety of Direct Hemoperfusion Using Polymyxin B-Immobilized Polystyrene Column for Patients With COVID-19: Protocol for an Exploratory Study
Source: JMIR Res Protoc. 2022 Nov 16;11(11):e37426. doi: 10.2196/37426 (PMC9674082; doi:10.2196/37426)

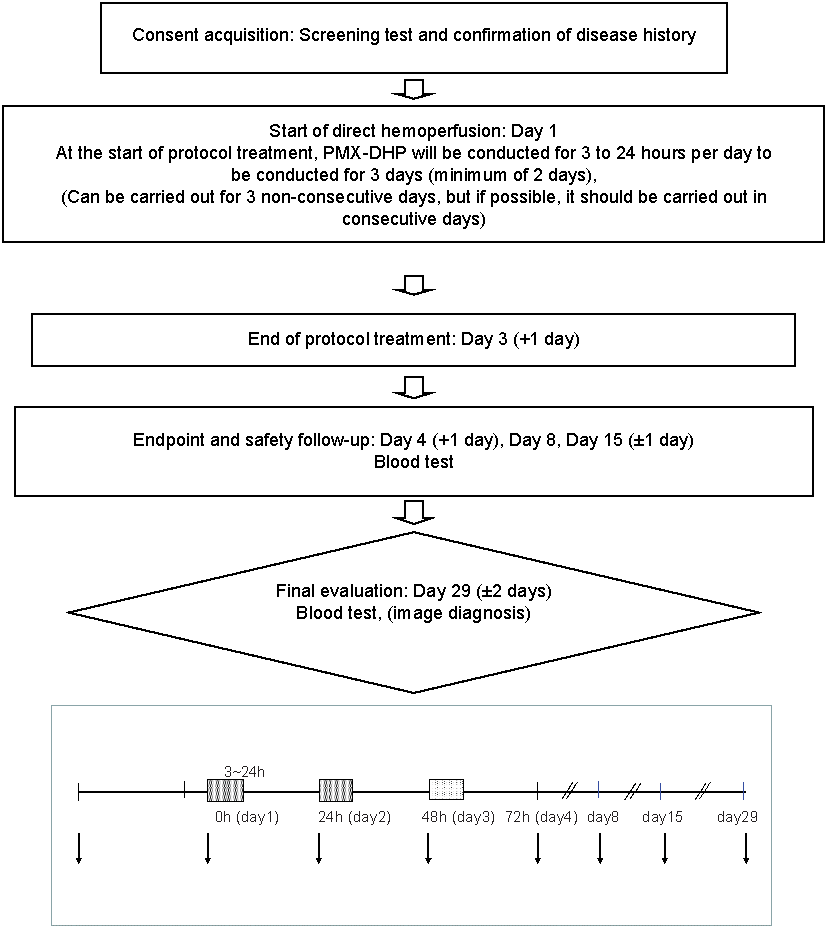

Supplement: Multimedia Appendix 1 [file resprot_v11i11e37426_app1.png]

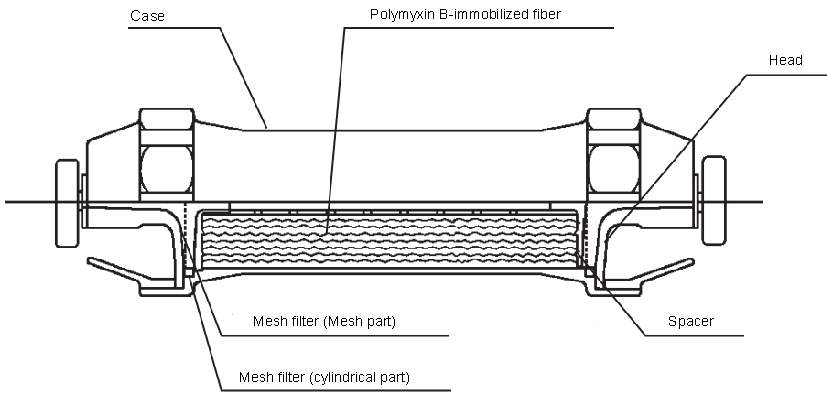

Supplement: Multimedia Appendix 2 [file resprot_v11i11e37426_app2.png]

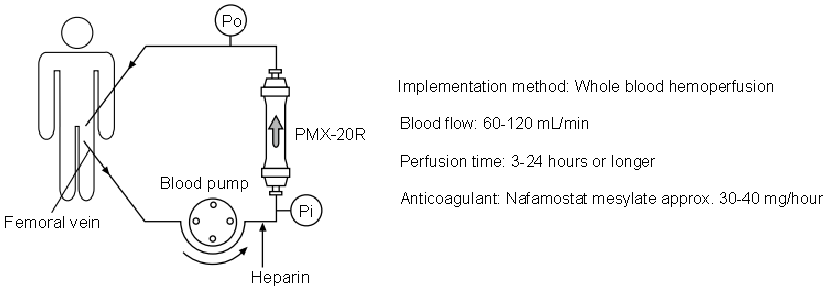

Supplement: Multimedia Appendix 3 [file resprot_v11i11e37426_app3.png]
